# Efficacy of Platelet-Rich Plasma in the Treatment of Equine Tendon and Ligament Injuries: A Systematic Review of Clinical and Experimental Studies

**DOI:** 10.3390/vetsci12040382

**Published:** 2025-04-18

**Authors:** Jorge U. Carmona, Catalina López

**Affiliations:** 1 Grupo de Investigación Terapia Regenerativa, Departamento de Salud Animal, Universidad de Caldas, Calle 65 No 26-10, Manizales 170004, Colombia; 2 Grupo de Investigación Patología Clínica Veterinaria, Departamento de Salud Animal, Universidad de Caldas, Calle 65 No 26-10, Manizales 170004, Colombia; catalina.lopez@ucaldas.edu.co

**Keywords:** platelet-rich plasma (PRP), equine tendon and ligament injuries, regenerative therapy, mesenchymal stem cells (MSCs), veterinary sports medicine

## Abstract

(1) Background: Tendon and ligament injuries are a leading cause of lameness in horses, with significant economic implications. Platelet-rich plasma (PRP) has gained attention for its regenerative potential, but its efficacy remains uncertain due to inconsistent study designs and reporting. (2) Methods: This systematic review, following the PRISMA guidelines, evaluated 22 studies (clinical and experimental) to assess the safety and efficacy of PRP in treating equine tendon and ligament injuries. The risk of bias was analyzed using the ROBINS-I and RoB 2.0 tools. (3) Results: PRP demonstrated a favorable safety profile, with no severe adverse effects reported. Clinical outcomes included improved lameness scores, ultrasonographic tissue organization, and return-to-work rates. However, variability in PRP formulations (e.g., leukocyte-rich vs. leukocyte-reduced) and activation methods (e.g., calcium chloride, thrombin) contributed to inconsistent results. Experimental studies supported PRP’s role in collagen synthesis and neovascularization, but comparative trials with stem cells or other therapies (e.g., extracorporeal shockwave) showed mixed results. The methodological quality of studies varied, with only 27% achieving “good” scores for PRP reporting. (4) Conclusions: PRP is a safe and potentially effective treatment, but its clinical application is hindered by a lack of standardization. Future research should focus on large, randomized controlled trials with uniform PRP protocols, long-term (≥2 years) efficacy assessments, comparative studies with MSC combinations, and cost-effectiveness analyses.

## Simple Summary

Tendon and ligament injuries frequently cause prolonged recovery and reduced performance in horses. Platelet-rich plasma (PRP) shows promise as a treatment, promoting healing while demonstrating excellent safety. This PRISMA-guided review of 22 studies found PRP that improves lameness, tissue healing, and return-to-competition rates. However, significant variability in preparation methods—including platelet/leukocyte concentrations, activation techniques, and dosing—hinders protocol optimization. While combining PRP with mesenchymal stem cells may enhance outcomes, more controlled trials are needed. The review emphasizes the critical need for standardized PRP production and reporting to ensure reproducible results. Current evidence supports PRP’s therapeutic potential, but larger, methodologically rigorous studies are required to establish definitive treatment guidelines. Future research should focus on standardizing PRP formulations, investigating combination therapies, and conducting long-term efficacy studies. These steps will help translate PRP’s promise into consistent clinical benefits for equine athletes.

## 1. Introduction

Soft tissue musculoskeletal disorders are common in horses and typically include acute traumatic injuries such as tendonitis and desmitis, as well as chronic or degenerative conditions such as tendinopathies and desmopathies. Acute lesions are often part of a chronic disease process and can be misinterpreted as isolated acute disorders. In many cases, what initially appears to be an acute injury may actually represent an episodic flare-up within a well-established clinicopathologic framework of chronic tendinopathy or desmopathy [1,2,3]. The most commonly affected soft tissue structures in the equine musculoskeletal system include the superficial digital flexor tendon (SDFT), deep digital flexor tendon (DDFT), and suspensory ligament (SL) [4].

Injuries to these structures account for approximately 10% or more of equine lameness cases [5] and represent a significant economic loss to the equine industry [6]. The exact causes of tendinopathy and desmopathy remain unclear, with multiple contributing factors, including aging, obesity, cumulative fatigue, and prior traumatic injuries, among others [1,7,8]. Interestingly, proinflammatory and catabolic cytokines such as interleukin-1 beta (IL-1β), tumor necrosis factor alpha (TNF-α), and interferon gamma (IFN-γ) have been immunolocalized in horses with tendinopathy but not in healthy horses [9]. This suggests that these mediators may play roles in perpetuating inflammation, disrupting the metabolic balance of the extracellular matrix, and inducing aberrant cell apoptosis in injured tendons and ligaments [1].

Traditionally, tendon and ligament injuries have been treated with a variety of medical, surgical, and physical therapy approaches aimed at alleviating pain, reducing inflammation, promoting healing, and restoring function [10]. However, in some cases, the healing process may result in fibrosis or scar formation [11].

Over the past 20 years, several regenerative medicine approaches using orthobiologics—such as bone marrow aspirate concentrates (BMACs), mesenchymal stem cells (MSCs), platelet-rich plasma (PRP), and related hemocomponents—have become increasingly popular for the treatment of joint and soft tissue injuries in horses [7,12,13]. One of the major beneficial effects claimed for orthobiologic products is their potent anabolic and anti-inflammatory paracrine effects mediated by growth factors (GFs) such as transforming growth factor beta 1 (TGF-β_1_), platelet-derived growth factors (PDGFs), vascular endothelial growth factor (VEGF), hepatocyte growth factor (HGF), insulin-like growth factors (IGFs), and others, as well as anti-inflammatory cytokines such as IL-4 and IL-10 [7,14]. When injected into injured tendons or ligaments, these mediators promote the inhibition of nuclear factor kappa B (NF-κB), thereby reducing inflammation, enhancing stem cell trafficking and differentiation, stimulating angiogenesis, and supporting extracellular matrix synthesis [15,16].

PRP is perhaps one of the simplest, most versatile, and cost-effective orthobiologics for treating tendon and ligament injuries in horses [17,18]. This hemocomponent is primarily considered a suspension of “live platelets and leukocytes” in plasma [19], with varying concentrations of these cells, depending on the method of preparation used to procure this orthobiologic. Consequently, PRP can be classified into different types based on the leukocyte concentration, including leukoreduced PRP (pure PRP; P-PRP) or leukocyte-rich PRP (L-PRP) [14]. P-PRPs are typically defined as blood-derived components containing low concentrations of platelets, with platelet counts comparable to or exceeding those found in whole blood. They also contain minimal to no leukocytes. In contrast, L-PRPs are characterized by the presence of measurable leukocyte levels and significantly higher platelet concentrations [20,21]. Once injected, PRP transforms into a platelet-rich gel (PRG), which initiates the release of several bioactive molecules, including GFs and anti-inflammatory/regulatory cytokines. This process creates an immunomodulatory hub that attracts nearby stem cells and promotes regenerative mechanisms, ultimately aiding in the repair and healing of damaged tissue [14].

The primary objective of this study was to systematically review the existing scientific literature regarding the clinical efficacy of PRP in the treatment of horses with clinical or experimentally induced tendon and ligament injuries. Specifically, this review aimed to answer several key questions: (1) What adverse effects have been reported with intralesional PRP injections in horses, and what safety profile can be established from the available studies? (2) What types of PRP are most commonly used to treat horses with tendon and ligament injuries? (3) What are the optimal concentrations of platelets and leukocytes in PRP for the treatment of these soft tissue musculoskeletal conditions? (4) Should PRP be activated prior to intralesional injection, and, if so, which platelet activator is most effective? (5) What is the recommended dosing regimen for PRP in the treatment of equine tenodesmic injuries? (6) What are the clinical outcomes of horses with tenodesmic injuries treated with PRP in the short term (1–3 months), medium term (3.1–11.9 months), and long term (1 year or more)? And, (7) Is the combined use of PRP with mesenchymal stem cells or bone marrow aspirates a better therapy for the treatment of tendon and ligament injuries in horses than the use of PRP alone?

## 2. Materials and Methods

This systematic review did not involve the use of living organisms or clinical or experimental procedures on animals. Therefore, it was not subject to the requirement of approval from an Ethics Committee for Animal Experimentation during its development.

### 2.1. Systematic Review Protocol

The methodology for this systematic review was designed in accordance with the guidelines outlined by PRISMA (Preferred Reporting Items for Systematic Reviews and Meta-Analyses) [22,23,24]. A search was conducted of the scientific literature regarding the use of PRP as a clinical treatment for horses with naturally occurring tendon and ligament injuries, experimental treatment of equine experimentally induced tendon and ligament injuries, or as intralesional injections (ILIs) in equine normal tendons or ligaments. This search was performed across three databases: Web of Science, Scopus, and PubMed. The time frame for article inclusion was from 1 January 2000 to 31 December 2024. The search terms used in these databases were (“platelet-rich plasma” OR “platelet concentrate*”) AND (horse* OR equine OR equus OR equid*) AND (tendonitis OR tendinopathy* OR “tendon injury*” OR desmitis OR desmopathy* OR “ligament injury*”).

#### 2.1.1. PICO Strategy

The six research questions addressed in this scoping review were based on the PICO framework (Population, Intervention, Comparison, Outcome) [25,26,27], as outlined below.

**P** was sport horses, working horses, or experimental horses. **I** was horses with naturally occurring tendonitis (tendinopathy) or desmitis (desmopathy), horses with experimentally-induced tendonitis (tendinopathy) or desmitis (desmopathy), or horses in which the safety and efficacy of ILIs of PRP was assessed in either normal tendons or ligaments. **C** was horses treated with alternative experimental (i.e., MSCs, bone marrow aspirates, and bone marrow mononuclear cells (BMMNCs), among others) or conventional therapies (i.e., corticosteroids, hyaluronan, and physical therapy, among others), a control group of horses, or those treated with ILIs of saline, phosphate-buffered saline, or Ringer’s lactate solutions. **O** was semiquantitative clinical parameters (e.g., lameness severity) and objective measurements such as ultrasound (US) examination, magnetic resonance image study, histology, plasma or urine biomarkers, hemogram, and others. The outcomes were also considered in the short term (1–3 months), medium term (1–11 months), and long term (1 year or more). Clinical studies without follow-up were not included. A high level of return to work was considered when horses were free of lameness in the long term.

#### 2.1.2. Eligibility Criteria for Publications and Protocol for Data Collection, Screening, and Inclusion

C.L. and J.U.C. independently accessed each database using the predefined keywords for this review. The articles identified were uploaded into an online reference management tool (EndNote Web, Clarivate, London, UK (https://www.myendnoteweb.com/EndNoteWeb.html) accessed on 14 February 2025) and duplicates were removed.

Each identified article was independently evaluated by the authors based on the title, keywords, and abstract. Articles were included if they met the following criteria: (1) articles focused on PRP in horses, particularly randomized clinical trials (RCTs), case series (CSs), and controlled experimental studies (CESs) published in scientific journals in all languages from 1 January 2000 to 31 December 2024; (2) treatment with any PRP preparation (P-PRP or L-PRP); (3) horses with naturally occurring tenodesmic injuries or experimental conditions; and (4) availability of full-text versions. The exclusion criteria included (1) narrative or systematic reviews, meta-analyses, commentaries, letters to the editor, abstracts, and individual case reports; (2) studies conducted in species other than horses; (3) research focused on pathologies in tissues unrelated to tendons and ligaments; and (4) articles without specific information, such as the number of horses treated or the time of follow-up. Following these criteria, the selected records were reviewed and analyzed by both authors. Disagreements regarding article inclusion were resolved through consensus.

### 2.2. Summarizing the Articles

Once reviewed, the selected articles were summarized into several tables, including one for cases series in which only PRP was used; one for case series in which PRP was used in combination with MSCs or other treatments; one for randomized clinical trials in which PRP was compared to saline or combined with MSCs, BMMCs or physical therapy; one for experimental studies in which PRP was evaluated alone; and one for experimental studies in which PRP was evaluated in combination with MSCs. The tables contained the following details: study (authors), study design, results, adverse effects, length of follow-up, and outcomes. The analysis included an evaluation of outcomes over the short, medium, and long terms, such as the ability of treated horses to return to sport or work, and the use of paraclinical data like ultrasonographic and magnetic resonance image findings or plasma or urine biomarker concentrations, among others. The clinical outcomes were categorized as positive, negative, or neutral. To enhance the clinical relevance of this review, the findings were discussed in the context of their practical implications for veterinary practitioners. For example, the efficacy of PRP in reducing the recovery time and improving functional outcomes in sport and working horses was highlighted as a key consideration for veterinarians managing equine tendon and ligament injuries.

### 2.3. Platelet-Rich Plasma Quality

The selected studies were analyzed using a modified set of criteria that outlined the essential information that PRP studies should report for an objective analysis and reproducibility of the results [28] to determine PRP quality. These criteria included 10 characteristics (Characteristic (C) C1, the source of blood whether autologous (AUT) or allogeneic (ALL); C2, the anticoagulant, volume, and age of the blood used to prepare PRP; C3, the method used to prepare the PRP; C4, the centrifugation conditions (*g* value, temperature, and time) used in the laboratory or in commercial PRP preparation devices; C5, a complete description of how the PRP was harvested (i.e., from buffy coats or PRP supernatants) and, if a commercial preparation device was used to include its commercial brand; C6, a measurement of the cellular content of the original whole blood, including the platelet count, white blood cell count, and red blood cell count; C7, a measure of the quality of the PRP preparation (i.e., cell content, platelet activation status, platelet-specific proteins, and growth factor content); C8, the concentration factor and yield of platelets obtained; C9, whether the PRP was activated prior to use, including the substance used to activate the platelets; and C10, the method and number of in vivo applications, the specific sites of application, and the volume of PRP administered) [28]. In addition, each characteristic was scored from 0 to 10 resulting in an overall score of 0 to 100. Studies with a score <40 were classified as having a poor methodological description of PRP, studies with a score between 41 and 80 were classified as having a moderate methodological description of PRP, and studies with a score >81 were classified as having a good methodological description of PRP.

### 2.4. Bias Risk Assessment

Each study’s potential bias was evaluated following the PRISMA guidelines [25,26,27]. Case series were evaluated according to the criteria stablished for assessing the risk of bias in non-randomized studies of interventions [29], based on seven domains: (1) bias due to confounding; (2) bias in the selection of participants into the study; (3) bias in the classification of interventions; (4) bias due to deviations from the intended interventions; (5) bias due to missing data; (6) bias in the measurement of outcomes; and (7) bias in the selection of the reported result. The documents that met the inclusion criteria were judged for each of the domains as having a low, moderate, serious, or critical risk of bias [29]. On the other hand, either randomized clinical or experimental studies were evaluated according to the criteria stablished for assessing risk of bias in randomized trials [30], according to five domains: (1) bias arising from the randomization process; (2) bias due to deviations from the intended interventions; (3) bias due to missing outcome data; (4) bias in the measurement of the outcome; and (5) bias in the selection of the reported result. The studies that met the inclusion criteria were judged for each of the domains as having a low, some concerns, or high risk of bias [30].

Disagreements between reviewers during the bias assessment process were resolved through consensus discussions to ensure consistency and reliability in the classification of bias levels. Examples of potential bias in the included studies were documented, such as inadequate randomization procedures, lack of blinding of outcome assessments, and incomplete reporting of clinical outcomes. In addition, the limitations of this review were acknowledged, including the heterogeneity among the included studies in terms of PRP preparation methods and outcome measures. These factors were taken into account when interpreting the overall results of this review. The bias assessment results were visualized using the ROVIS tool and the ROBINS-I and RoB2.0 datasets (https://mcguinlu.shinyapps.io/robvis/, accessed on 14 February 2025) [31].

## 3. Results

### 3.1. General Results

**Figure 1:**
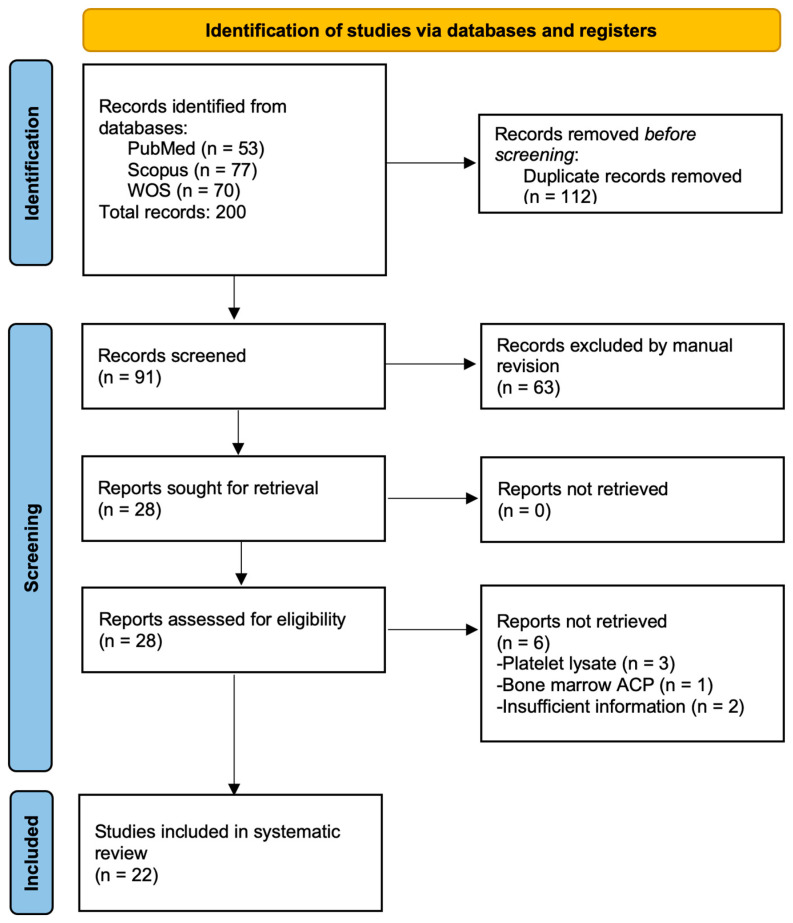
Flowchart of the study selection process according to PRISMA.

The search strategy identified a total of 200 articles across three major databases: PubMed (n = 53), SCOPUS (n = 77), and Web of Science (n = 70). Following removal of 112 duplicate records, we retained 91 unique articles for preliminary screening. Through a rigorous evaluation of titles, abstracts, and keywords, we excluded 63 articles based on the following criteria: (1) irrelevant population—studies focusing on non-equine species or non-musculoskeletal conditions (e.g., human tendon injuries) and (2) intervention mismatch—investigations of therapies other than PRP (e.g., stem cell-only treatments). After a full-text review of the remaining 28 articles, we ultimately included 22 studies that met all eligibility criteria (Figure 1).

The six articles excluded from the review were three articles that evaluated the effect of platelet lysates [32,33], one study that used a PRP kit to concentrate a bone marrow aspirate [34], and two studies had insufficient information [35,36].

Of the twenty-two selected records, five case series [17,18,37,38,39] evaluated the effects of PRP on horses with tendinopathies and desmopathies (Table 1). Four case series [40,41,42,43] evaluated PRP in combination with mesenchymal stem cells, bone marrow, or bone marrow mononuclear cells under similar conditions (Table 2). Additionally, four randomized clinical trials [44,45,46,47] evaluated PRP either alone or in combination with other therapies for tendinopathies and desmopathies (Table 3). Seven controlled experimental studies [48,49,50,51,52,53,54] investigated the effects of PRP on these conditions (Table 4), while two experimental studies [55,56] assessed PRP in combination with mesenchymal stem cell therapies (Table 5). Overall, no severe adverse effects were reported in the evaluated studies. The included studies varied widely in sample size, ranging from small case series (e.g., five horses in Argüelles et al. [18]) to larger clinical trials (e.g., one hundred horses in Giunta et al. [46]). Notably, 12/22 studies (54.5%) had sample sizes ≤ 10 horses, while only 4/22 (18.2%) included ≥50 horses.

The diagnosis and evolution of the tendon and ligament lesions were performed by a subjective lameness examination and US evaluation. These diagnostic approaches were mainly used in two case series [18,37] and three clinical trials [44,45,47]. Conversely, a lameness evaluation was not a primary diagnostic tool in most experimental models of induced lesions surgically or with collagenase prior to PRP application [48,49,50,51,52,53,54].

In general, a US evaluation was combined with a clinical lameness assessment to correlate structural and functional improvements. All studies used a US evaluation to confirm and monitor tendon and ligament healing. High-frequency US imaging was used for lesion size measurements [39,42] and assessments of collagen fiber organization [50,51]. The monitoring of neovascularization was performed by color Doppler US (CDUS) [49]. Histologic and molecular diagnostic methods were used primarily in most experimental models of surgically or collagenase-induced lesions prior to PRP application to assess tissue remodeling at the cellular level. Histology was used to evaluate the collagen fiber orientation and density [52,54], fibroblast proliferation, and matrix remodeling [55,56]. Furthermore, immunohistochemistry for Factor VIII was used to assess vascularization and endothelial activity [48,54], while a gene expression analysis (COL1A1, tenascin, and MMPs) was performed to assess tenogenic differentiation and tissue regeneration potential [56].

In studies where PRP was used as the sole clinical treatment, improvements were observed in lameness, ultrasound (US) appearance, and return-to-competition times [17,18,37,38,39]. In some cases, PRP promoted tissue healing but did not fully restore normal ultrasound features, particularly in chronic hindlimb PSLD cases [18]. Randomized trials demonstrated that PRP enhanced lameness reduction and matrix organization; however, long-term return-to-work rates were comparable to those achieved with extracorporeal shockwave therapy (EST) [46] or bone marrow aspirate concentrate (BMAC) [47] treatments. On the other hand, in experimental models, PRP increased collagen production, the glycosaminoglycan (GAG) content, and cellularity [50]; stimulated neovascularization and blood flow; improved fibrillogenesis; and reduced inflammation [48,49]. Histological studies confirmed enhanced collagen fiber alignment, although fibroblast and vascular responses varied [52,54].

PRP was scored as positive in all case series studies [17,18,37,38,39,40,41,42,43], in two [44,45] out of four randomized clinical trials, in six [48,49,50,51,52,53] (85.7%) out of seven experimental studies in which PRP was used as the only treatment, and in one [55] of out two experimental studies in which PRP was evaluated together mesenchymal stem cells. In general, 18 (82%) of out 22 studies were scored with positive outcome, 2 [46,47] (9%) of out 22 studies were scored with negative outcome, and 2 [54,56] (9%) out of 22 studies had a neutral outcome.

Where reported, the magnitude of improvement in PRP-treated groups varied widely: lameness scores decreased by 30–60% (vs. 10–25% in controls) in RCTs [44,45], while ultrasonographic fiber alignment improved by 40–80% in experimental studies [48,49,50]. Return-to-work rates ranged from 70–89% for PRP vs. 50–60% for saline controls [17,39,45], though these differences were not always statistically significant due to small sample sizes.

**Table 1:** Case series in which the effect of various platelet-rich plasma (PRP) preparations was evaluated on horses with tendon and ligament injuries.

Study	Study Type, Number of Animals, and Objectives	Study Design	Results and Adverse Effects	Length of Follow-Up	Outcome
Argüelles et al. [18]	Case series; 5 horses, 2 with superficial digital flexor tendon tendinopathy (SDFTT) and 3 with proximal desmitis of the suspensory ligament (PDSL). Objective: To evaluate the effect of autologous platelet-rich plasma (PRP) on lameness scores and ultrasound (US) changes.	4–5 mL of PRP was injected intralesionally (IL) 3 times at 2-week intervals; US and lameness evaluations were performed prior to each treatment.	Improvements in lameness and US appearance in SDFTT cases. Clinical improvement but no US changes in PDSL cases. No side effects were reported.	20 months	Positive
Castelijns et al. [37]	Case series; 11 horses with 18 suspensory ligament (SL) branch (SLB) injuries. To evaluate the effect of an autologous PRP product on lameness scores and US changes.	2.5 mL of PRP obtained by a gravitational method was injected IL only once per affected structure. Horses were evaluated by a lameness examination and US before and 3 months after treatment	A good US appearance was noted in 8 horses at 3 months. Ten horses were not lame after 3 months. Five horses returned to full work, 1 to a lower level, 3 retired, 1 was still recovering, and 1 died of unrelated causes at 3 years. No side effects were reported.	36 months	Positive
Georg et al. [38]	Case series; 7 horses, 5 with SDFTT, 1 with inferior check ligament injury, and 1 with tendinopathy of the deep digital flexor tendon (DDFTT). To evaluate the effect of an autologous PRP product (autologous conditioned plasma (ACP)) on lameness scores and US changes.	2–4 mL of PRP obtained by a semiautomated kit was injected IL only once per affected structure. Horses were evaluated by a lameness examination and US before and 2 and 3 weeks after treatment.	In general, all horses showed improvement of the US appearance at 3 weeks after treatment. All horses returned to the previous workload or full training at 9 months. No adverse effects were reported.	10–13 months	Positive
Waselau et al. [17]	Case series; 9 horses with midbody SL desmitis (MSD). The aim was to evaluate the effect of one IL PRP dose by US examination and follow-up of race performance over three years.	7–12 mL PRP was injected IL, followed by controlled exercise. Horses were monitored by US.	A total of 100% returned to racing between 28 and 68 weeks. Nine horses raced during first and second years, while only five raced during the third year. No adverse effects were reported.	36 months	Positive
Zuffova et al. [39]	Case series; 22 horses with SDFTT. The aim was to evaluate the effectiveness of PRP in treating SDFT lesions by US and return to racing.	The PRP volume was injected IL according to the size of the lesion. Only one PRP dose was injected.	PRP shortened the rehabilitation time, and increased the return to racing. No adverse effects.	Up to 48 months	Positive

**Table 2:** Case series in which the effects of various PRP preparations combined with mesenchymal stem cells (MSCs) and bone marrow mononucleated cells (BMMNCs) were evaluated in horses with tendon and ligament injuries.

Study	Study Type, Number of Animals, and Objectives	Study Design	Results and Adverse Effects	Length of Follow-Up	Outcome
Beerts et al. [40]	Case series; 104 horses. The aim was to evaluate the combination of allogeneic mesenchymal stem cells (MSCs) and PRP by US and lameness examinations in 68 horses with SLD and 36 with SDFTT.	One IL injection of 2–3 × 10^6^ allogeneic MSCs (1 mL) plus 1 mL of PRP. Horses were evaluated at 6 and 12 weeks, and 12 and 24 months.	At 24 months, 82.4% of horses with SLD and 85.7% with SDFTT maintained competition levels. Some horses (14.7% with SL and 14.3% with SDFT) relapsed and retired. No major adverse effects were reported.	24 months	Positive
Guercio et al. [41]	Case series; 9 horses with SDFTT. The aim was to evaluate the combination of adipose-derived MSCs (AD-MSCs) plus PRP by US and clinical follow-up.	One only dose of 1 × 10^6^ autologous AD-MSCs mixed with 5–10 mL of PRP was injected IL. Horses were evaluated by US at 1, 2, and 4 months.	Seven horses resumed their normal competitive activity after 9 months. Two horses were reinjured. No adverse effects.	9 months	Positive
Ricco et al. [42]	Case series; 19 horses with SDFTT. The aim was to evaluate the effect of allogeneic AD-MSCs in combination with autologous PRP by US and performance results.	One only dose of 2–6 mL of PRP containing 2 × 10^6^ AD-MSCs/mL was injected IL.	US improvement at 3 months. Seventeen horses (89.5%) returned to competition, while two (10.5%) were reinjured. No adverse effects were reported.	24 months	Positive
Torricelli et al. [43]	Case series; 13 horses, 12 with SLD and 1 with SDFTT. The aim was to evaluate the effect of autologous bone marrow mononuclear cells (BMMNCs) plus PRP by US, lameness examination, and performance results.	One only dose of 4–7 mL of BMMNCs (1.9 × 10^6^ cells) and autologous PRP was injected IL.	All horses showed an US improvement. Eleven horses (84.6%) returned to competition. Faster recovery was observed in horses treated with PRP with more than 750 × 10^3^/µL platelets (2.8 ± 0.4 months) with respect to horses who received less concentrated PRP (7.9± 4.3 months). No adverse effects.	12 months	Positive

The acronyms are the same as in Table 1.

**Table 3:** Randomized clinical studies evaluating the effects of PRP preparations either alone or combined with cells or physical therapy on horses with tendon and ligament injuries.

Study	Study Type, Number of Animals, and Objectives	Study Design	Results and Adverse Effects	Length of Follow-Up	Outcome
Garrett et al. [44]	Randomized clinical trial; 39 horses with proximal sesamoiditis and associated SL branch desmitis (SLBD). The aim was to compare the effects of PRP and a saline solution (SS) by US and performance results.	Horses were randomized to receive a single 3 mL dose of PRP (treatment group) or 3 mL of SS (control group). Racing performance was followed for 2, 3, and 4 years.	PRP horses more likely to race at 2 years old. No significant difference in racing performance was observed at 3 and 4 years.	36 months	Positive
Geburek et al. [45]	Randomized controlled clinical trial; 20 horses with SDFTT. The aim was to compare the effects of PRP and SS by US and performance results.	Horses were randomized to receive a single 3 mL dose of PRP (treatment group) or 3 mL of SS (control group). US and clinical evaluations were performed at 5 intervals up to 24 weeks.	The PRP group showed an earlier reduction in lameness and improved matrix organization. No significant difference in the cross-sectional area was observed. A total of 80% of PRP horses returned to the previous or higher level vs. 50% of control horses at 12 months.	24 months	Positive
Giunta et al. [46]	Prospective randomized trial; 100 horses with PSD. The aim was to compare PRP with extracorporeal shockwave therapy (EST) by US, lameness examination, and performance results.	Horses were randomly allotted to receive 3–6 mL of autologous PRP or EST (800 pulses). The PRP group included 47 horses, 19 with affected forelimbs and 28 with affected hindlimbs. The EST group included 49 horses, 14 with affected forelimbs and 35 with affected hindlimbs. Horses were evaluated at 4 days, 6 months, and 12 months.	EST improved short-term lameness more than PRP. At one year, EST had a higher return-to-work rate overall. PRP showed better outcomes in horses with severe ultrasound changes. EST associated with a 3.8x higher return to work at 1 year.	12 months	Negative
Maleas and Mageed [47]	Randomized controlled multicentric clinical trial; 93 horses with chronic hindlimb PSLD. The aim was to compare the effect of autologous PRP with a bone marrow aspirate concentrate (BMAC) by clinical, US, and performance results.	Horses were allocated into 3 groups, Control (exercise only, n = 22), 2–4 mL of PRP (n = 46), and 2–4 mL of BMAC (n = 25), and evaluated at 6, 12, and 18 months.	BMAC yielded better lameness scores than PRP; 84% of BMAC horses were sound. Both BMAC and PRP were significantly better than controlled exercise alone for treating chronic hindlimb PSD. BMAC appeared to lead to better short- and long-term outcomes (soundness) than PRP. The cytology of BMAC did not predict outcomes. No adverse effects were reported.	18 months	Negative

The acronyms are the same as in Table 1 and Table 2.

**Table 4:** Randomized experimental studies evaluating the effects of various platelet-rich plasma (PRP) preparations on horses with tendon and ligament injuries.

Study	Study Type, Number of Animals, and Objectives	Study Design	Results and Adverse Effects	Length of Follow-up	Outcome
Bosch et al. [50]	Experimental, placebo-controlled study of 6 clinically healthy horses. The effect of PRP on standardized, surgically induced lesions in the forelimb SDFTs was evaluated at 24 weeks from treatment by biochemical, biomechanical and histology analyses.	Surgically induced forelimb SDFT lesions were randomly treated with a single dose of 3 mL of autologous PRP or SS. Horses were maintained on an exercise protocol, and euthanasia was performed at 24 weeks.	The PRP group had significantly higher collagen, GAG, and DNA contents. An improved tensile strength and elastic modulus were observed in the PRP group. Histology revealed a better structural organization and higher metabolic activity in the PRP group.	24 weeks	Positive
Bosch et al. [48]	Experimental, placebo-controlled study of 6 clinically healthy horses. The aim was to evaluate the effect of PRP on neovascularization by color Doppler US (CDUS) and immunohistochemistry in surgically induced lesions in the forelimb SDFTs.	Surgically induced forelimb SDFT lesions were randomly treated with a single dose of 3 mL of autologous PRP or SS. Horses were maintained on an exercise protocol, and euthanasia was performed at 24 weeks. Limbs were scanned at 1 week after surgery, just before the PRP or placebo injection, and at 2, 3, 5, 8, 12, 18 and 24 weeks after surgery. Tendon samples were prepared for an immunohistochemical analysis of Factor VIII.	The PRP group showed significantly higher blood flow and vessel formation than the placebo group, except at week 5. Factor VIII staining confirmed more organized vascular structures.	24 weeks	Positive
Bosch et al. [49]	Experimental, placebo-controlled study of 6 clinically healthy horses. The aim was to evaluate the effect of PRP by an analysis of computerized ultrasound images and histology in surgically induced lesions in the forelimb SDFTs.	Surgically induced forelimb SDFT lesions were randomly treated with a single dose of 3 mL of autologous PRP or SS. Horses were maintained on an exercise protocol, and euthanasia was performed at 24 weeks. Limbs were scanned at 1 week after surgery, just before the PRP or placebo injection, and at 2, 3, 5, 8, 12, 18 and 24 weeks after surgery.	The PRP group showed superior healing, with an 80% collagen orientation compared to 60% for the placebo group. PRP improved fibrillogenesis and structural organization and reduced inflammation. The ultrasonographic analysis confirmed accelerated tendon repair, supporting PRP as an effective treatment.	24 weeks	Positive
Figueiredo et al. [51]	Experimental, placebo-controlled study of 6 clinically healthy horses. The effect of PRP on standardized, surgically induced lesions in fore- and hindlimb SLs. The aim was to evaluate the effect of PRP using US, morphological, and morphometric analyses of the repaired tissue.	Surgically induced SL lesions were randomly treated with 2.5 mL of PRP or SS. US was performed on days 10, 30, and 60. On day 46, biopsies of scar tissue were taken for a histological analysis.	PRP-treated suspensory ligament lesions showed improved healing with improved echogenicity, increased neovascularization, and increased type I collagen levels.	46 days	Positive
Maia et al. [53]	Experimental, placebo-controlled study of 6 clinically healthy horses. The aim was to evaluate the effect of PRP on collagenase-induced tendinitis by a US examination.	Tendinitis was induced in the forelimb SDFTs by a collagenase injection. PRP (2.5 mL) or saline was applied. The US evaluation was performed at 48 h and days 7, 12, 14, 21, 28, 35, and 42.	The PRP group showed a greater lesion reduction, improved echogenicity, and better collagen fiber alignment compared to controls. US assessments at multiple time points confirmed smaller lesion cross-sectional areas in the PRP group.	42 days	Positive
Maia et al. [52]	Experimental, placebo-controlled study of 6 clinically healthy horses. The aim was to evaluate the effect of PRP on collagenase-induced tendinitis by a histology analysis.	Tendinitis was induced in the forelimb SDFTs by a collagenase injection. PRP (2.5 mL) or saline was applied. After 36 days, biopsy and histology assessed the fibroblast density, neovascularization, and tissue organization.	The PRP group showed a better collagen fiber alignment, higher fibroblast density, and moderate neovascularization. Histology revealed more organized tissue repair in PRP-treated tendons, although fibroblast and vessel counts were not significantly different from controls.	36 days	Positive
Zandim et al. [54]	Experimental, placebo-controlled study of 6 clinically healthy horses. The aim was to evaluate the effect of PRP on collagenase-induced tendinitis by histologic and morphometric changes and immunohistochemistry of Factor VIII expression.	Tendinitis was induced in the forelimb SDFTs by a collagenase injection. PRP (1.8 mL) or saline was applied. Biopsies were taken at 3 and 16 days for histologic, morphometric, and immunohistochemical analyses.	No significant differences were found between PRP- and saline-treated tendons in collagen organization, fibroblast density, vascularization, or Factor VIII expression. PRP did not accelerate tendon healing, highlighting the need for further studies on its efficacy.	16 days	Neutral

The acronyms are the same as in Table 1, Table 2 and Table 3.

**Table 5:** Randomized experimental studies evaluating the effects of PRP preparations combined with MSCs on horses with tendon injuries.

Study	Study Type, Number of Animals, and Objectives	Study Design	Results and Adverse Effects	Length of Follow-Up	Outcome
Carvalho et al. [55]	Experimental study of 8 horses. The aim was to evaluate the effects of AD-MSCs) suspended in PRP on collagenase-induced forelimb SDFT tendonitis using ultrasound, histopathology, immunohistochemistry, and gene expression analyses.	Forelimb SDFT lesions were treated with 10 × 10^6^ AD-MSCs suspended in 1 mL autologous PRP or SS (control). US monitoring was performed every two weeks for 16 weeks, followed by biopsy and histologic and molecular analyses.	AD-MSC + PRP prevented lesion progression, improved collagen fiber organization, and reduced inflammation. US showed improved healing in treated tendons. Gene expression levels for collagen and tenogenic markers were not significantly different between groups.	16 weeks	Positive
Romero et al. [56]	Experimental study of 12 horses. The aim was to compare the effects of BM-MSCs, AD-MSCs, and PRP on surgically induced SDFT lesions using ultrasound, histology, and gene expression analyses.	Lesions were treated with BM-MSCs (20 × 10^6^ cells suspended in 7 mL of lactated Ringer’s solution (LRS)), AD-MSCs (20 × 10^6^ cells suspended in 7 mL of LRS), PRP (7 mL), or the control (7 mL of LRS). Healing was monitored by ultrasound at 2, 6, 10, 20, and 45 weeks, followed by histopathology and a molecular analysis.	BM-MSCs showed an earlier improvement in echogenicity (week 6), while all treatments outperformed controls by week 10. PRP and AD-MSCs showed similar healing results, both improving ultrasound echogenicity and outperforming controls. Histology confirmed improved collagen fiber alignment in all treated tendons, with BM-MSCs showing superior effects compared to PRP and AT-MSCs. The gene expression analysis revealed increased levels of COL1A1, tenascin, and matrix metalloproteinases in BM-MSC-treated tendons, suggesting enhanced tissue regeneration. PRP and AT-MSCs showed a moderate upregulation of tenogenic markers, although less than BM-MSCs.	45 weeks	Neutral

The acronyms are the same as in Table 1, Table 2, Table 3 and Table 4.

### 3.2. Platelet-Rich Plasma Quality

Fresh autologous whole blood was used as the source of PRP in all but one study [40]. The anticoagulants used for PRP procurement were sodium citrate (SC) in 8/22 (36.4%) studies [18,41,43,50,52,53,54,56], acid citrate dextrose (ACD) in 4/22 reports [17,37,44,45] (18.17%), and citrate phosphate dextrose (CPD) in 3/22 registries [39,40,42] (13.50%), while no anticoagulant was reported in 8/22 (36.35%) studies [38,46,47,48,49,50,51,55]. The volume of fresh whole blood (WB) collected across the studies for PRP preparation ranged from 15 mL to 300 mL, with most studies using 50–81 mL [17,18,37,38,39,43,44,45,47,52,53]. However, 9/22 (40.90%) of the studies did not report the volume of WB used to produce PRP [42,46,48,49,50,51,54,56]. Most studies (10/22, 45.45%) used double centrifugation tube protocols to obtain PRP products [18,41,42,43,51,52,53,54]; 8/22 (36.36%) studies used single centrifugation semi-automated kits [17,38,44,45,46,48,49,50,55]; 2/22 (9.09%) studies used a gravitational filter system [37,47]; while 1/22 (4.54%) studies used a multiple centrifugation tube step protocol to obtain PRP [40].

Most studies (14/22, 63.63%) did not report WB basal platelet and leukocyte concentrations [18,40,41,42,46,47,48,49,50,51,52,53,54,55,56], while 4/22 (18.18%) studies reported only WB basal platelet concentrations [17,39,43,44] and 4/22 studies reported WB basal platelet and leukocyte concentrations [37,38,45,54]. The platelet concentration in PRP was reported in all studies but one [46]. Platelet concentrations in PRP ranged from 100 × 10^3^ to 1370 × 10^3^ PLTs/µL, with most studies achieving between a 1.3- to 8.7-fold increase over baseline WB platelet counts. However, data for platelet enrichment in PRP was reported only in 9/22 (40.90%) studies [17,37,38,39,43,44,45,48,50]. On the other hand, only 9/22 (40.90%) studies reported the concentration of leukocytes in PRP [37,38,40,45,48,50,51,55,56]. In these studies, the leukocyte concentration in PRP ranged from 0.1 × 10^3^ to 42.1 × 10^3^ leukocytes/µL [40,48,50], with five studies [37,48,50,56] classifying PRP as L-PRP and four studies [38,40,45,51,55] classifying PRP as P-PRP [20,21].

Quality measures of PRP, such as a growth factor concentration determination, were reported only in 4/22 (18.18%) studies [18,43,48,50]. The main mediators measured were TGF-β_1_, PDGF, VEGF, and IGF. On the other hand, 11/22 (50%) studies did not report whether PRP was activated or not at the time of its therapeutic or experimental application [37,38,40,41,42,46,47,48,49,50,55]. PRP was injected without any activating agent in five studies [44,45,51,54,56], activated with calcium chloride in five studies [18,39,43,52,53], and activated with bovine thrombin in one study [17]. Of note, sodium bicarbonate was added to PRP as a buffer in one study [44]. PRP was injected intralesionally in all the studies. The volume of PRP administered ranged from 1 mL to 12 mL, with most studies using 2–5 mL per injection. Most studies administered a single injection, although Argüelles et al. [18] used three injections at 2-week intervals. PRP was combined with stem cells and related cells in 5/22 (22.71%) studies [40,41,42,43,55]. A summary of the 22 studies analyzed in this review according to the 11 characteristics of the Harrison and Alsousou criteria [28] is presented in Table 6, Table 7, Table 8, Table 9 and Table 10.

**Table 6:** Characteristics of platelet-rich plasma (PRP) used as the only treatment in the case series according to the Harrison and Alsousou criteria.

	Characteristic (C) *									
Study	C1	C2	C3	C4	C5	C6	C7	C8	C9	C10
Argüelles et al. [18]	AUT	SC, 75 mL of fresh WB	Double centrifugation tube method	120× *g*/5 min, then 240× *g*/5 min. Room temperature	PRP collected from the buffy coat.	NR	250 ± 71.8 × 10^3^ PLTs/µL; TGF-β_1_: 1251 pg/ml	NR	Calcium chloride	3 intralesional (IL) injections of 5–8 mL of PRP at 2-week intervals
Castelijns et al. [37]	AUT	ACD, 55 mL of fresh WB	Gravitational filter system	NA	E-PET set, Pall Corporation filter, NY, USA. Platelets were captured via a filter and back-flushed with harvest solution. L-PRP	92.8 ± 3.5 × 10^3^ PLTs/µL; 5.6 ± 1.3 ×10^3^ WBCs/µL	648 ± 312.6 × 10^3^ PLTs/µL; 21.4 ± 5.2 WBCs ×10^3^/µL. L-PRP	6.9 ± 1.9-fold increase	NR	2.5 mL of PRP IL; 1 injection
Georg et al. [38]	AUT	15 mL of WB	Semiautomated kit. Single centrifugation	NR	ACP double syringe Arthrex, Germany. PRP was collected from the buffy coat. P-PRP	144 × 10^3^ PLTs/µL; 8.71 × WBCs 10^3^/µL	178.5 × 10^3^/PLTs µL; 1.01 × 10 ^3^ WBCs/µL. P-PRP	1.3-fold increase	NR	2–4 mL of PRP IL, with 1–2 injections
Waselau et al. [17]	AUT	ACD, 50 mL of WB	Semiautomated kit. Single centrifugation	2100× *g*/9 min	Secquire, PPAI Medical, FL, USA. PRP was collected from the buffy coat.	155 × 10^3^ PLTs/µL	1370 × 10^3^ PLTs/µL	8.7-fold increase	Bovine thrombin	7–12 mL of PRP IL; 1 injection
Zuffova et al. [39]	AUT	CPDA, 50 mL of WB	Single centrifugation tube method	1500 rpm/5 min	PRP was collected from the buffy coat.	87.8 ± 18.5 × 10^3^ PLTs/µL	544.8 ± 244.2 × 10^3^ PLTs/µL	5.6-fold increase	Calcium chloride	The PRP volume was relative to the size of the lesion; 1 dose IL

* C1: The source of blood, whether autologous (AUT) or allogeneic (ALL). C2: The anticoagulant, volume, and age of the blood used to prepare PRP. C3: The method used to prepare the PRP. C4: The centrifugation conditions (g value, temperature, and time) used in the laboratory or in commercial PRP preparation devices. C5: A complete description of how the PRP was harvested (i.e., from buffy coats or PRP supernatants) and, if a commercial preparation device was used, to include its commercial brand. C6 A measurement of the cellular content of the original whole blood, including the platelet count, white blood cell count, and red blood cell count. C7. A measure of the quality of the PRP preparation (i.e., cell content, platelet activation status, platelet-specific proteins, and growth factor content). C8. The concentration factor and yield of platelets obtained. C9. Whether the PRP was activated prior to use, including the substance used to activate the platelets. C10. The method and number of in vivo applications, the specific sites of application, and the volume of PRP administered. ACD, acid citrate dextrose; CPDA, citrate phosphate dextrose adenine; NA, not applicable; NR, not reported; SC, sodium citrate; WB, whole blood.

**Table 7:** Characteristics of PRP used in combination with mesenchymal stem cells (MSCs) and bone marrow mononucleated cells (BMMNCs) as a treatment in the case series according to the Harrison and Alsousou criteria.

	Characteristic (C)									
Study	C1	C2	C3	C4	C5	C6	C7	C8	C9	C10
Beerts et al. [40]	ALL	CPDA-1, 300 mL of WB	Multiple centrifugation steps	NR	PRP was collected from the buffy coat. P-PRP	NR	100–150 × 10^3^ PLTs/µL, 0.1 × 10^3^ WBCs/µL. P-PRP	NR	NR	1 mL of PRP/allogeneic MSCs; 1 injection
Guercio et al. [41]	AUT	SC, 250 mL of WB	Double centrifugation	180× *g* for 10 min, then 1200× *g* for 10 min	Platelets were collected from the buffy coat.	NR	1000 × 10^3^ platelets/µL	NR	NR	5–10 mL of PRP/AD-MSCs; 1 injection IL
Ricco et al. [42]	AUT	CPD	Double centrifugation in 50 mL tubes.	150× *g*/30 min, then 800× *g*/15 min at 4 °C	The platelet pellet was collected after centrifugation.	NR	1000 × 10^3^ PLTs/µL	NR	NR	2–6 mL of PRP with 2 × 10^6^ ASCs/mL
Torricelli et al. [43]	AUT	SC, 50 mL of WB	Double centrifugation	200× *g*/5 min, then 1000× *g*/15 min	PRP was collected from the buffy coat.	144 × 10^3^ PLT/µL	751 × 10^3^ PLTs/µL; TGF-β1, 3055 pg/mL; PDGF-AB, 357.1 pg/mL; VEGF, 169.1 pg/mL; IGF, 289.2 pg/mL; EGF, 4.6 pg/mL; and IL-1β, 3.9 pg/mL	5.4-fold increase	Calcium chloride	4–7 mL of PRP/BMMNCs; 1 injection IL

The acronyms are the same as in Table 1, Table 2, Table 3, Table 4, Table 5 and Table 6.

**Table 8:** Characteristics of PRP preparations (either alone or combined with other therapies) in randomized clinical studies according to the Harrison and Alsousou criteria.

	Characteristic (C)									
Study	C1	C2	C3	C4	C5	C6	C7	C8	C9	C10
Garrett et al. [44]	AUT	ACD, 55 mL of WB	Semiautomated kit. Single centrifugation	1744× *g*/15 min	PRP was collected from the buffy coat. GPS II Platelet Concentrate Separation Kit, Biomet Inc, IN, USA	214 ± 113 × 10^3^ PLTs/µL	966 ± 189 × 10^3^ PLTs/µL	5.2-fold increase	Non-activated	3 mL of PRP or saline; 1 dose IL
Geburek et al. [45]	AUT	ACD-A, 54 mL of WB	Semiautomated kit. Double centrifugation	900× *g*/3 min, then 1470× *g*/10 min	PRP was collected from the buffy coat. Osteokine^®^, PRP preparation system, Orthogen, Düsseldorf, Germany	157.3 ± 35.9 × 10^3^ PLTs/µL; 7.5 ± 1.5 × 10^3^ WBCs/µL	892.37 ± 364.7 × 10^3^ PLTs/µL; 14.1 ± 7.0 × 10^3^ WBCs/µL. L-PRP	5.67-fold increase	Non-activated	3 mL of PRP or saline; 1 dose IL
Giunta et al. [46]	AUT	NR	Semiautomated kit.	NR	Arthrex ACP system, FL, USA	NR	NR	NR	NR	3–6 mL of PRP; 1 dose IL
Maleas and Mageed [47]	AUT	55 mL of WB	Gravitational filter system	NR	E-PET set, Pall Corporation filter, NY, USA. L-PRP	NR	495 ± 364.7 × 10^3^ PLTs/µL	NR	NR	2–4 mL of PRP; 1 dose IL

The acronyms are the same as in Table 1, Table 2, Table 3, Table 4, Table 5, Table 6 and Table 7.

**Table 9:** Characteristics of PRP used in the experimental studies according to the Harrison and Alsousou criteria.

	Characteristic (C)									
Study	C1	C2	C3	C4	C5	C6	C7	C8	C9	C10
Bosch et al. [48]	AUT	SC	Semiautomated kit. Single centrifugation	NR	PRP was collected from the buffy coat. GPS II Biomet, IN, USA	NR	639.7 ± 103.2 × 10^3^ PLTs/µL; 42.1 ± 16.7 × 10^3^ WBCs/µL; TGF-β, 4810.9 ± 1530.5 pg/mL; PDGF-, 5000 ± 830 pg/mL. L-PRP	3.78-fold increase	NR	Single injection, 3 mL of PRP per tendon
Bosch et al. [49]	AUT	NR	Semiautomated kit. Single centrifugation	NR	PRP was collected from the buffy coat. GPS II Biomet, IN, USA	NR	NR	NR	NR	Single injection, 3 mL of PRP per tendon
Bosch et al. [50]	AUT	NR	Semiautomated kit. Single centrifugation	NR	PRP was collected from the buffy coat. GPS II Biomet, IN, USA	NR	639.7 ± 103.2 × 10^3^ PLTs/µL; 42.1 ± 16.7 × 10^3^ WBCs/µL; TGF-β, 4810.9 ± 1530.5 pg/mL; PDGF-, 5000 ± 830 pg/mL. L-PRP	3.78-fold increase	NR	Single injection, 3 mL of PRP per tendon
Figueiredo et al. [51]	AUT	NR	Double centrifugation tube method	120× *g*/5 min, then 240× *g*/5 min	PRP was collected from the buffy coat.	NR	300× 10^3^ PLTs/µL; 2.0 × 10^3^ WBCs/µL. P-PRP	NR	Non-activated	Single injection, 2.5 mL of PRP
Maia et al. [52]	AUT	SC, 81 mL of WB	Double centrifugation method	120× *g*/5 min, then 473× *g*/5 min	PRP was collected from the buffy coat.	NR	300–500 × 10^3^ PLTs/µL	NR	Calcium chloride	Single injection, 2.5 mL of PRP per tendon
Maia et al. [53]	AUT	SC, 81 mL of WB	Double centrifugation method	120× *g*/5 min, then 473× *g*/5 min.	PRP was collected from the buffy coat.	NR	300–500 × 10^3^ PLTs/µL	NR	Calcium chloride	Single injection, 2.5 mL of PRP per tendon
Zandim et al. [54]	AUT	SC	Double centrifugation method	120× *g*/5 min, then 240× *g*/5 min	PRP was collected from the buffy coat.	164.5 ± 9.89 ×10^3^ PLTs/µL	368.3 ± 39.7 ×10^3^ PLTs/µL	NR	Non-activated	Single injection, 1.8 mL of PRP per tendon

The acronyms are the same as in Table 1, Table 2, Table 3, Table 4, Table 5, Table 6, Table 7 and Table 8.

**Table 10:** Characteristics of PRP combined with MSCs in the experimental studies according to the Harrison and Alsousou criteria.

	Characteristic (C)									
Study	C1	C2	C3	C4	C5	C6	C7	C8	C9	C10
Carvalho et al. [55]	AUT	NR	Double centrifugation tube method	120× *g*/5 min, then 240× *g*/5 min	NR	NR	321 × 10^3^ PLTs/µL; 2.3 × 10^3^ WBCs/µL. P-PRP	NR	NR	Single injection, 1 mL of PRP/MSCs
Romero et al. [56]	AUT	SC	Double centrifugation tube method	120× *g*/5 min, then 240× *g*/5 min	PRP was collected from the buffy coat.	NR	263.3 ± 99.9 ×10^3^ PLTs/µL; 8.9 ± 2.5 × 10^3^ WBCs/µL. L-PRP	NR	Non-activated	Single injection, 7 mL of PRP per tendon

The acronyms are the same as in Table 1, Table 2, Table 3, Table 4, Table 5, Table 6, Table 7, Table 8 and Table 9.

The semiquantitative analysis used to classify the methodological description of PRP production and quality in the studies showed that the scores ranged from 40 [46,49] to 94 [45], with a median score of 67.5. Two [46,49] (9.09%) out of twenty-two studies presented a poor methodological description of PRP production and quality. Fourteen [18,38,40,41,42,47,48,50,51,52,53,54,55,56] (63.63%) out of twenty-two studies showed a moderate methodological description of PRP, while six [17,37,39,43,44,45] (27.27%) out of twenty-two studies had a good methodological description of PRP of production and quality (Table 11).

**Table 11:** Scoring of the studies according to the qualification of each characteristic according to the modified Harrison and Alsousou criteria.

			Characteristic (C)										Overall Score
Study	Type of Study	Treatment	C1	C2	C3	C4	C5	C6	C7	C8	C9	C10	
Argüelles et al. [18]	Case series	Only PRP	10	10	10	10	10	0	7	0	10	10	77
Castelijns et al. [37]	Case series	Only PRP	10	10	10	10	10	10	7	10	0	10	87
Georg et al. [38]	Case series	Only PRP	10	7	10	0	10	10	7	10	0	10	74
Waselau et al. [17]	Case series	Only PRP	10	10	10	10	10	5	3	10	10	10	88
Zuffova et al. [39]	Case series	Only PRP	10	10	10	5	10	5	3	10	10	10	83
Beerts et al. [40]	Case series	PRP/MSCs	10	10	10	0	10	0	7	0	0	10	57
Guercio et al. [41]	Case series	PRP/AD-MSCs	10	10	10	7	10	0	3	0	0	10	60
Ricco et al. [42]	Case series	PRP/AD-MSCs	10	3	10	10	10	0	3	0	0	10	56
Torricelli et al. [43]	Case series	PRP/BMMNCs	10	10	10	7	10	5	7	10	10	10	89
Garrett et al. [44]	RCT	PRP or SS	10	10	10	7	10	5	3	10	10	10	85
Geburek et al. [45]	RCT	PRP or SS	10	10	10	7	10	10	7	10	10	10	94
Giunta et al. [46]	RCT	PRP or EST	10	0	10	0	10	0	0	0	0	10	40
Maleas and Mageed [47]	RCT	PRP or BMAC	10	7	10	0	10	0	3	0	0	10	50
Bosch et al. [50]	RES	Only PRP	10	3	10	0	10	0	10	10	0	10	63
Bosch et al. [48]	RES	Only PRP	10	0	10	0	10	0	0	0	0	10	40
Bosch et al. [49]	RES	Only PRP	10	0	10	0	10	0	10	10	0	10	60
Figueiredo et al. [51]	RES	Only PRP	10	0	10	7	10	0	7	0	10	10	64
Maia et al. [53]	RES	Only PRP	10	10	10	7	10	0	3	0	10	10	70
Maia et al. [52]	RES	Only PRP	10	10	10	7	10	0	3	0	10	10	70
Zandim et al. [54]	RES	Only PRP	10	3	10	7	10	5	3	0	10	10	68
Carvalho et al. [55]	RES	PRP/MSCs	10	0	10	7	10	0	7	0	0	10	54
Romero et al. [56]	RES	PRP/MSCs	10	3	10	7	10	0	7	0	10	10	67

RCT, randomized clinical trial; RES, randomized experimental study. Other acronyms are the same as in Table 1, Table 2, Table 3, Table 4, Table 5, Table 6, Table 7, Table 8, Table 9, Table 10 and Table 11.

### 3.3. Bias Risk Assessment

**Figure 2:**
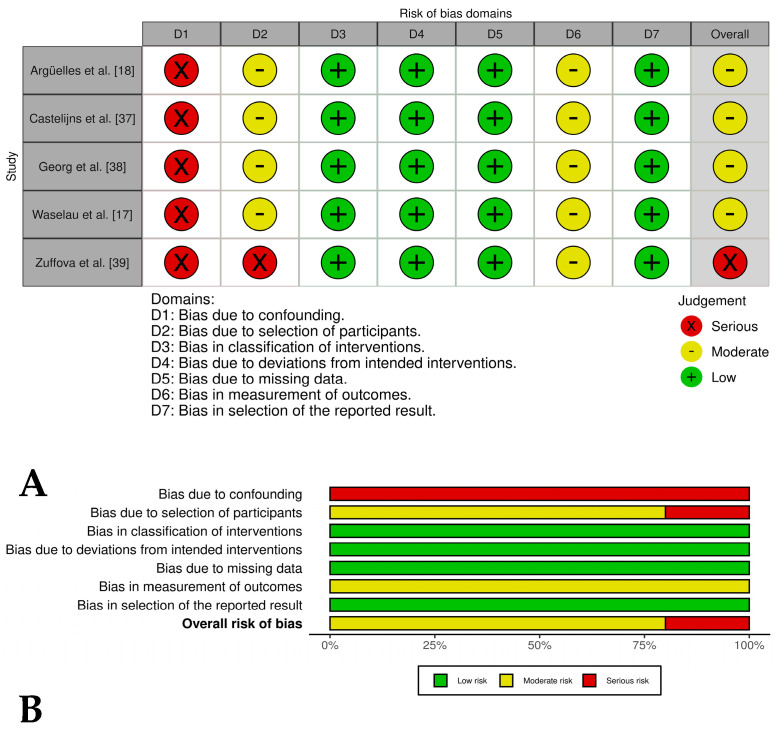
(**A**) Traffic light plots of the domain-level judgments for each study. Each study is color-coded (green, yellow, or red) to indicate the risk of bias across various domains. (**B**) Weighted bar plots showing the distribution of risk of bias ratings within each domain for case series of horses treated only with PRP. The bars represent the proportions of studies judged according to each domain.

Four out of five of the case series studies [17,18,37,38] evaluating PRP as only treatment for tendon and ligament injuries showed an overall moderate risk of bias due to deficiencies in three domains: confounding, selection of participants, and measurement of outcomes. On the other hand, one case series study [33] showed a serious risk of bias in the selection of participants domain, resulting in an overall serious risk of bias for this study (Figure 2A,B).

**Figure 3:**
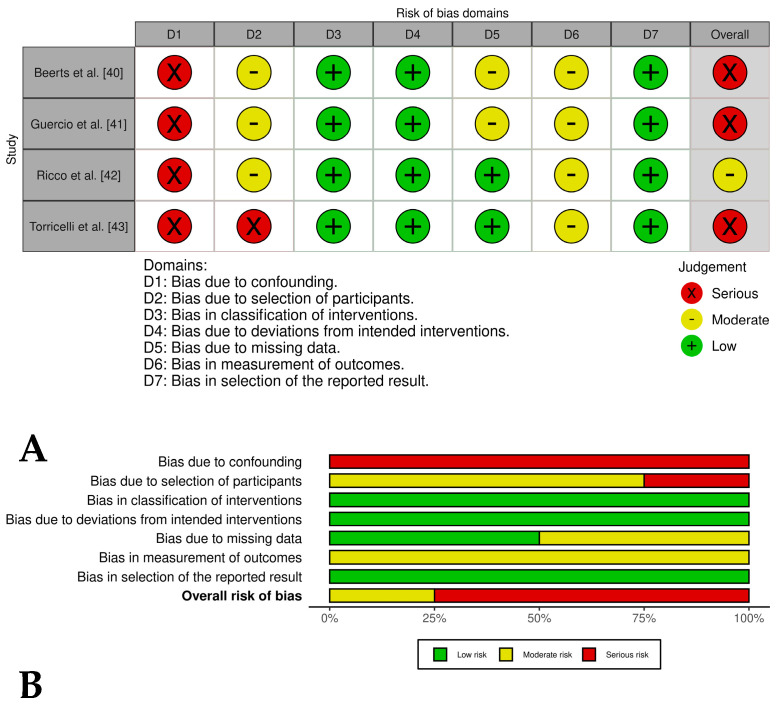
(**A**) Traffic light plots of the domain-level judgments for each study. Each study is color-coded to indicate the risk of bias across various domains. (**B**) Weighted bar plots showing the distribution of risk of bias ratings within each domain for case series of horses treated with PRP plus stem cells or related cells. The bars represent the proportions of studies judged according to each domain.

Three [40,41,43] of out of four case series in which PRP was evaluated in combination with stem cells and related cells showed an overall serious risk of bias, while one only study [42] presented an overall moderate risk of bias. The most critical domains judged were confounding, selection of participants, and missing data (Figure 3A,B). In general, the two case series groups evaluated presented a lack of control groups, unclear selection procedures, and small, heterogeneous samples that introduced potential bias; a lack of blinding of clinicians or owners may have influenced post-treatment care and outcomes; and subjective outcomes (i.e., lameness scores) were assessed without blinding, risking detection bias. Despite these limitations, the studies reported positive results, including reduced lameness and high rates of return to work.

**Figure 4:**
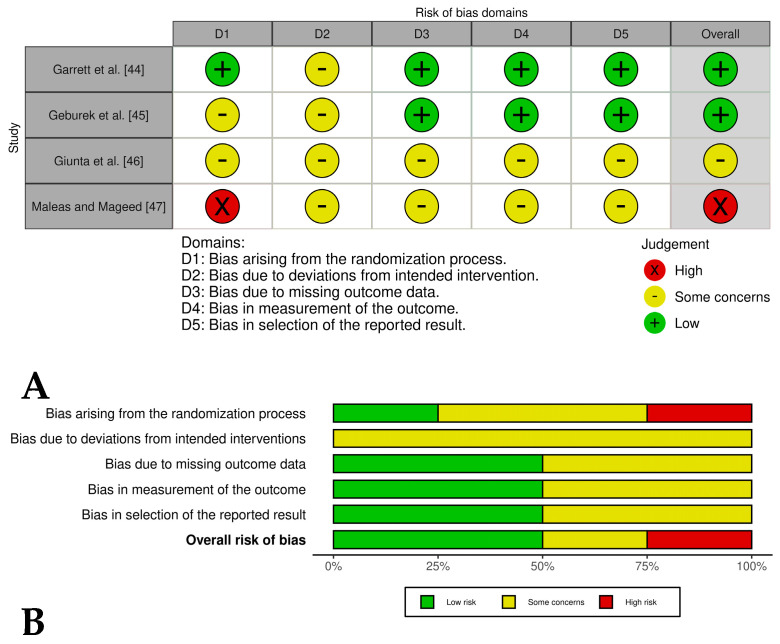
(**A**) Traffic light plots of the domain-level judgments for each study. Each study is color-coded to indicate the risk of bias across various domains. (**B**) Weighted bar plots showing the distribution of risk of bias ratings within each domain for controlled clinical trials of horses treated with PRP plus another therapies. The bars represent the proportions of studies judged according to each domain.

**Figure 5:**
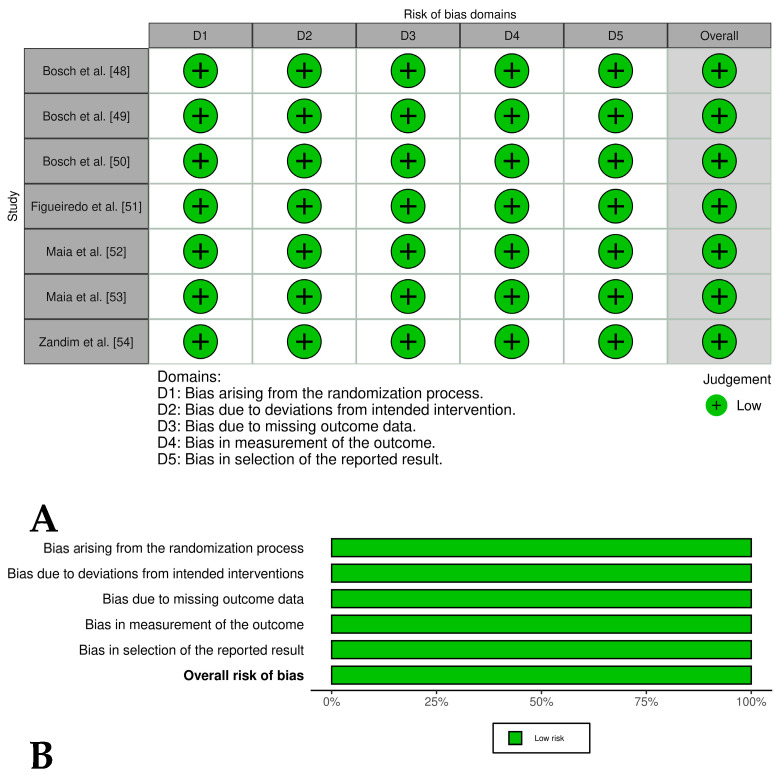
(**A**) Traffic light plots of the domain-level judgments for each study. Each study is color-coded to indicate the risk of bias across various domains. (**B**) Weighted bar plots showing the distribution of risk of bias ratings within each domain for experimental studies of horses treated only with PRP. The bars represent the proportions of studies judged according to each domain.

**Figure 6:**
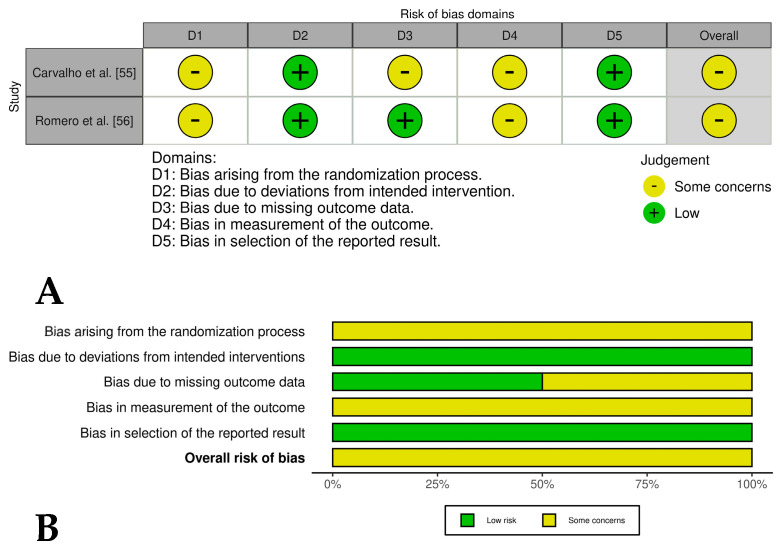
(**A**) Traffic light plots of the domain-level judgments for each study. Each study is color-coded to indicate the risk of bias across various domains. (**B**) Weighted bar plots showing the distribution of risk of bias ratings within each domain for experimental studies of horses treated with PRP and mesenchymal stem cells. The bars represent the proportions of studies judged according to each domain.

Two [44,45] of the four included controlled clinical trials had an overall low risk of bias, while one study [46] had an overall risk of bias of some concern and another study [47] had an overall high risk of bias. These last studies had problems such as a lack of detailed randomization and allocation concealment, unblinded treatment administration, and potential selective reporting due to lack of pre-registered protocols (Figure 4A,B). The randomized experimental studies [48,49,50,51,52,53,54] evaluating the isolated effect of PRP showed a low risk of bias across all domains, indicating high methodological quality and the reliability of results (Figure 5A,B), while the two randomized experimental trials evaluating the combination of PRP with mesenchymal stem cells [55,56] showed an overall risk of bias of some concerns (Figure 6A,B).

## 4. Discussion

The results of this systematic review highlight the growing body of evidence supporting the use of PRP in the treatment of tendon and ligament injuries in horses. Among studies with a low risk of bias and robust methodology (e.g., refs. [44,45,48,49,50]), PRP demonstrated consistent improvements in lameness scores, ultrasonographic appearance, and return-to-competition rates compared to untreated controls. However, these findings were less conclusive in studies with a moderate-to-high bias risk, underscoring the need for a cautious interpretation. On the other hand, PRP has been found to promote tissue healing, stimulate neovascularization, and improve collagen fiber alignment, particularly in experimental models [48,49,50,51,52,53]. However, despite these positive findings, there are significant limitations that must be considered, particularly regarding the study design, PRP preparation protocols, and variability in outcome measures.

The results of this systematic review strongly support the safety of intralesional PRP injections in horses. No significant adverse effects such as infection or severe inflammation were reported following PRP administration in the 22 studies reviewed. This suggests that PRP is a well-tolerated treatment option for equine tendon and ligament injuries. The safety profile of PRP is further supported by experimental studies in which no evidence of cytotoxicity, excessive fibrosis, or adverse immune responses were observed [48,49,50,51,52,53,54]. However, the lack of standardized criteria for reporting mild adverse events or transient inflammatory responses is a limitation that should be addressed in future clinical trials.

The studies reviewed lacked sufficient information on the concentration of leukocytes in PRP. Only nine of the twenty-two studies [37,38,40,45,48,50,51,55,56] reported the concentrations of these cells, allowing these PRPs to be classified as L-PRP, and four studies [38,40,51,55] classified PRP as P-PRP [20,21]. In general, the clinical outcomes of these studies were positive, although one study [56] had a neutral result using an L-PRP preparation. The information obtained from the analysis of these studies does not allow a conclusion as to which type of PRP (either P-PRP or L-PRP) may be more appropriate for the treatment of tendon and ligament injuries in horses. These findings may contradict a previous in vitro study of equine tendons, where P-PRP may be the optimal preparation to stimulate superior healing without scar tissue formation [57]. This highlights the urgent need for comparative studies that specifically evaluate the influence of the leukocyte concentration on PRP efficacy, particularly under clinical conditions.

Significant heterogeneity in platelet and leukocyte concentrations was observed among the twenty-two studies reviewed, with only nine [37,38,40,45,48,50,51,55,56] reporting specific leukocyte counts. This variability makes it difficult to draw definitive conclusions regarding the optimal platelet and leukocyte concentrations in PRP for the treatment of equine tendon and ligament injuries. In addition, two studies with negative clinical outcomes reported conflicting results: one used a semi-automated kit to prepare P-PRP [46], while the other used a semi-automated kit to prepare L-PRP [47]. These results suggest that the concentrations of platelets and leukocytes may not be the only critical factors influencing treatment efficacy. Instead, other aspects, such as the appropriate selection of patients for PRP therapy, may play a more important role in determining clinical outcomes. This underscores the need for standardized protocols and further research to better understand the factors that contribute to the success of PRP treatments for equine musculoskeletal injuries. In addition, future studies should investigate the role of patient-specific factors such as age, breed, and injury severity in determining the optimal PRP formulation and treatment protocol.

Half of the studies included in this review did not report whether PRP was activated before use or specify the activation method. However, good clinical and experimental outcomes were observed in studies where PRP was either not activated or activated using calcium chloride [18,39,43,52,53] or bovine thrombin [17]. These findings suggest that the activation of PRP may not be essential to enhance its therapeutic effects when injected into injured tendons and ligaments. However, the data from this systematic review do not provide sufficient evidence to draw definitive conclusions regarding the necessity of using activators for PRP in the treatment of equine tendon and ligament disorders. In particular, the activators commonly used in these studies, such as calcium chloride and bovine thrombin, have been associated with potential drawbacks, including crystal deposition (calcium chloride) and inflammatory reactions (bovine thrombin) in treated tissues [58,59,60]. This raises concerns about their suitability for clinical use. In contrast, calcium gluconate, which does not precipitate into crystalline forms in PRP clots, has been proposed as a more favorable alternative [58]. Further research is needed to evaluate the efficacy and safety of various activation methods, including non-activated PRP, to optimize treatment protocols for equine musculoskeletal injuries. In addition, future studies should explore alternative activation methods, such as the use of autologous thrombin or mechanical activation, to minimize potential adverse effects.

The dosing regimen for PRP varied widely across studies, with injection volumes ranging from 1 mL to 12 mL per treatment. The majority of studies used a single intralesional injection, while one study used multiple injections administered at 2-week intervals, particularly for chronic injuries [18]. The volume of PRP administered was often adjusted based on the size and severity of the lesion, suggesting a tailored approach to treatment. Although single injections have demonstrated efficacy in many cases, repeated administrations may provide an additional benefit for more severe or chronic conditions [18,38]. However, the lack of standardized dosing protocols highlights the need for further research to establish evidence-based guidelines. Notably, in all case series and randomized clinical trials, PRP administration was combined with controlled exercise protocols implemented to enhance tissue adaptation and promote functional recovery. This combination underscores the importance of integrating PRP therapy with rehabilitation strategies to optimize the clinical outcomes of equine tendon and ligament injuries. Future research should also investigate the optimal timing and frequency of PRP injections, especially in chronic or recurrent injuries, to maximize the therapeutic benefits.

Short-term outcomes (1–3 months) showed that PRP treatment was associated with significant improvements in lameness scores and ultrasound appearance in most clinical studies [37,38,42,43,45]. Medium-term outcomes (3.1–11.9 months) showed continued improvements in tissue healing and functional recovery, with many horses returning to previous levels of activity [17,38,41]. Long-term outcomes (1 year or more) were more variable, with some studies reporting sustained improvements [18,37,39,40,42] and others noting a decline in performance, particularly in horses with chronic cases [17,40,42,44]. Overall, PRP appears to be effective at promoting short- and medium-term recovery, but its long-term efficacy may depend on factors such as the injury severity and concurrent therapies. Further research into the durability of PRP effects over time is needed to determine its role in long-term tendon and ligament management. Moreover, future studies should include longer follow-up periods (e.g., 2–5 years) to assess the durability of PRP treatment and its impact on preventing reinjury.

The combined use of PRP with MSCs (either from bone marrow or adipose tissue), BMMNCs, or BMAC showed promising results in several studies [40,41,42,43,55], demonstrating enhanced tissue regeneration and improved long-term outcomes. However, no randomized clinical trials or experimental studies have directly compared the effects of PRP alone, stem cells alone, or their combination to confirm a potential synergistic effect. Controlled clinical trials evaluating PRP in combination with MSCs or other regenerative products are essential to validate their potential additional benefits and to develop optimized treatment protocols. Future research should also explore the use of other regenerative therapies, such as extracellular vesicles [61,62,63] or gene therapy [63], in combination with PRP to further enhance tissue repair and regeneration.

To the authors’ knowledge, this is the first study to critically evaluate PRP preparation procedures and quality-related parameters using the modified Harrison and Alsousou criteria, which assess 10 key characteristics [28]. While some aspects of this discussion have been addressed in previous sections, the most important findings relate to the semi-quantitative scoring system used to assess the quality of information for each characteristic and the overall methodological rigor of the studies. Specifically, 72.72% of the reviewed studies demonstrated a poor to moderate methodological description of PRP production and quality, potentially limiting the reliability of their reported clinical outcomes. For example, studies that reported negative clinical outcomes [46,47] also had low semi-quantitative scores of 40 and 50 (Table 11), highlighting the impact of methodological inconsistencies on outcome interpretation. At this point, a consideration that this problem has been widely documented in several systematic reviews conducted in several human medicine fields [64], including orthopedics [65], is necessary. To address this issue, future studies should adhere to standardized reporting or criteria, such as those used in this study [28], or use other guidelines, such as the Minimum Information for Studies Evaluating Biologics in Orthopedics (MIBO) guidelines [66], to improve the quality and reproducibility of PRP research.

We used two risk of bias assessment tools to evaluate the studies included in this systematic review [31]: the ROBINS-I tool for non-randomized intervention trials [29] and RoB2.0 for randomized trials [30]. Overall, case series had a high risk of bias, mainly due to the lack of control groups and lack of blinding during interventions. This problem was particularly evident in studies evaluating PRP in combination with stem cells and other cellular products [40,41,43], as well as in one study evaluating PRP as a single treatment [39]. While the overall clinical outcomes in these studies were positive, their results should be interpreted with caution due to the methodological limitations identified in the risk of bias analysis. Future studies should prioritize the inclusion of control groups and blinding of both clinicians and owners to minimize bias and improve the reliability of the results.

Regarding the risk of bias analysis of randomized clinical trials, studies with an overall score of “some concerns” [46] and a “high risk” [47] of bias reported negative clinical outcomes for PRP. Notably, these studies also had lower scores (40 and 50) for the methodological description of PRP production and quality. In contrast, the randomized clinical trials by Garrett et al. [44] and Geburek et al. [45] evaluating PRP versus saline for the treatment of ligament and tendon injuries had a low overall risk of bias. These studies also achieved higher methodological scores for PRP production and quality, with scores of 85 and 94, respectively, further supporting their reliability. These findings highlight the importance of a rigorous study design and standardized reporting in PRP research to ensure the validity and reproducibility of the results.

The seven randomized experimental studies that evaluated the effects of PRP versus saline on equine models of tendinitis and desmitis [48,49,50,51,52,53,54] had an overall low risk of bias. However, their major limitations included short follow-up periods (ranging from 16 days to 24 weeks) and the absence of subjective or objective lameness assessments during the trials. In contrast, the two randomized experimental studies evaluating PRP combined with MSCs [55] and the isolated effects of BM-MSCs, AD-MSCs, PRP, and Ringer’s solution [56] had an overall risk of bias rated as “some concerns”. These concerns stemmed from problems in the randomization process, missing animals, and a lack of blinding of operators, which may have affected the reliability of their results. Future experimental studies should include longer follow-up periods and include both subjective (e.g., lameness scores) and objective (e.g., biomechanical testing) outcome measures to provide a more comprehensive evaluation of PRP efficacy.

This systematic review has several important limitations. The high heterogeneity in PRP preparation methods (e.g., centrifugation protocols, leukocyte concentrations, and activation techniques), study designs, and outcome measures precluded a meaningful meta-analysis or subgroup analyses [67]. Without standardized protocols or consistent methodologies, a quantitative synthesis would likely yield unreliable results, highlighting the need for more uniform research approaches in this field.

The predominance of small-scale studies (median of n = 9 horses) represents another significant constraint. Only three trials [40,46,47] included more than 50 subjects, increasing the risk of overestimated treatment effects and underreported adverse events. The neutral outcomes reported by some studies [54,56] may reflect insufficient statistical power rather than true therapeutic equivalence.

Additional limitations include (1) the high risk of bias in case series, particularly for long-term outcomes; and (2) the absence of cost-effectiveness data comparing PRP to conventional treatments. Future research should prioritize large, randomized trials with standardized protocols and incorporate economic analyses to address these gaps.

## 5. Conclusions

PRP demonstrates an excellent safety profile across studies, although its therapeutic value remains uncertain due to inconsistent evidence quality. Future research should (1) prioritize controlled efficacy trials over safety studies, (2) adopt standardized outcome measures, and (3) report negative results to avoid publication bias.

While our review suggests potential benefits of PRP, the predominance of small, methodologically heterogeneous studies with a moderate-to-high bias risk tempers the certainty of these findings. For instance, the positive outcomes reported in case series may reflect selection bias or placebo effects, as these studies lacked controls. Conversely, the most robust evidence (from randomized trials and controlled experiments) supports PRP’s efficacy but with modest effect sizes.

The variability in PRP preparation methods, including platelet and leukocyte concentrations, activation protocols, and dosing regimens, underscores the need for standardized protocols. While PRP shows promise as a regenerative therapy, its long-term (24 months or more) efficacy remains uncertain. Combining PRP with MSCs or other regenerative therapies may provide additional benefits, but more randomized controlled trials are needed to confirm this hypothesis. Standardized reporting and the methodological rigor of PRP studies need to be improved to ensure reproducibility and facilitate evidence-based veterinary practice.

## Data Availability

The original contributions presented in the study are included in the article; further inquiries can be directed to the corresponding author.

## Abbreviations

The following abbreviations are used in this manuscript:

ACD	Acid citrate dextrose
ACP	Autologous conditioned plasma
AD-MSCs	Adipose-derived mesenchymal stem cells
ALL	Allogeneic
AUT	Autologous
BMMNCs	Bone marrow mononuclear cells
CDUS	Color Doppler ultrasound
COL1A1	Collagen type I alpha 1 chain
CPDA	Citrate phosphate dextrose adenine
DDFT	Deep digital flexor tendon
EGF	Epidermal growth factor
EST	Extracorporeal shockwave therapy
GFs	Growth factors
GAG	Glycosaminoglycan
HGF	Hepatocyte growth factor
IFN-γ	Interferon gamma
IGFs	Insulin-like growth factors
IL-1β	Interleukin-1 beta
IL-4	Interleukin-4
IL-10	Interleukin-10
IL	Intralesional
L-PRP	Leukocyte and platelet-rich plasma
MMPs	Matrix metalloproteinases
MSD	Midbody suspensory ligament desmitis
NF-κB	Nuclear factor kappa B
PDGFs	Platelet-derived growth factors
PICO	Population, intervention, comparison, outcome
PLT	Platelet
PRG	Platelet-rich gel
PRISMA	Preferred Reporting Items for Systematic Reviews and Meta-Analyses
PRP	Platelet-rich plasma
P-PRP	Pure platelet-rich plasma
PSLD	Proximal suspensory ligament desmitis
ROBINS-I	Risk of bias in non-randomized studies of interventions
RoB2.0	Risk of bias 2 (tool for randomized trials)
ROVIS	Risk-of-bias VISualization tool
SC	sodium citrate
SDFT	Superficial digital flexor tendon
SDFTT	Superficial digital flexor tendon tendinopathy
SL	Suspensory ligament
SLBD	Suspensory ligament branch desmitis
SLD	Suspensory ligament desmitis
TGF-β_1_	Transforming growth factor beta 1
TNF-α	Tumor necrosis factor alpha
US	Ultrasound
VEGF	Vascular endothelial growth factor
WB	Whole blood
WBC	White blood cell
